# LncRNA LOC100129620 promotes osteosarcoma progression through regulating CDK6 expression, tumor angiogenesis, and macrophage polarization

**DOI:** 10.18632/aging.203042

**Published:** 2021-05-18

**Authors:** Yong Chen, Guoqing Tang, Hongbin Qian, Ji Chen, Bing Cheng, Chengliang Zhou, Yixin Shen

**Affiliations:** 1 Orthopedic Center, The Second Affiliated Hospital of Soochow University, Suzhou, China; 2 Kunshan Hospital of Traditional Chinese Medicine, Kunshan 215300, China; 3 Institute of Translational Medicine, Medical College, Yangzhou University, Yangzhou 225001, China

**Keywords:** osteosarcoma, long non-coding RNA, miR-335-3p, CDK6, proliferation

## Abstract

Osteosarcoma is a malignant tumor with high mortality in children and adolescents. The mechanism of osteosarcoma metastasis is currently unclear. Abnormal expression of long non-coding RNA (lncRNA) plays an important role in tumor metastasis. We used bioinformatics to analyze the differences in gene expression between osteosarcoma *in situ* and osteosarcoma lung metastases. CCK-8 was used to detect the effect of lncRNA LOC100129620 on the proliferation of osteosarcoma cells. The effect of LOC100129620 on the invasion of osteosarcoma cells was assessed by Transwell assay. The regulatory effect of LOC100129620 on miR-335-3p was examined using RNA pull-down and luciferase reporter gene assays. The effect of LOC100129620 on the polarization of macrophages was detected by quantitative real-time fluorescent PCR. The results show that LOC100129620 can promote the proliferation and migration of osteosarcoma cells. LOC100129620 can promote the proliferation of osteosarcoma *in vivo*. LOC100129620 can bind to miR-335-3p and regulate its function. MiR-335-3p mediates the regulatory effects of LOC100129620 on CDK6. LOC100129620 promotes the formation of blood vessels and the polarization of macrophages. The LOC100129620/miR-335-3p/CDK6 signaling pathway promotes the metastasis of osteosarcoma by regulating the proliferation of osteosarcoma cells, angiogenesis, and macrophage polarization.

## INTRODUCTION

Osteosarcoma often occurs in children and adolescents [1]. It is a primary malignant bone tumor that spreads extremely rapidly [2]. It is common in the metaphyses of long bones, such as the distal femur, proximal tibia, and proximal humerus [3]. Among all osteosarcoma patients, adolescents aged 10–20 years account for about 60%, and adults over 40 years account for about 13% [1]. Osteosarcoma in the elderly is often accompanied by osteitis deformans or secondary tumors. At present, surgical treatment is mainly used in the clinic, and combined treatment with radiotherapy, chemotherapy, and biological treatment can also be provided, but most treatments are not effective [4]. Although amputation can save the lives of some patients to a certain extent, for young patients, amputation not only causes serious damage to their bodies but also causes mental damage [5]. Although neoadjuvant chemotherapy combined with surgical treatment can improve the 5-year survival rate of patients, when osteosarcoma relapses and distant metastasis occurs, its prognosis is very poor. Therefore, more and more scholars are exploring the occurrence and development of osteosarcoma and the molecular biology of osteosarcoma.

Long non-coding RNA (lncRNA) is a type of functional RNA molecule with a transcript length of more than 200 nucleotides. Early studies found that it lacks conservative open reading frames (ORFs), so it was considered “noise” without biological characteristics [6]. However, scholars have discovered that lncRNA plays a key role in epigenetic regulation, transcriptional regulation, post-transcriptional regulation, and nuclear transport [7]. It is involved in histone modification, chromatin remodeling, DNA methylation, and gene activation or silencing [8]. At present, the role of lncRNA in the occurrence and metastasis of osteosarcoma has not been elucidated [9]. Transcriptome sequencing technology is widely used in the screening of tumor-related lncRNA expression profiles. A common method is to carry out high-throughput sequencing and screening of differentially expressed lncRNAs in tumor tissues and normal tissues, and then compare differentially expressed genes through bioinformatics to establish a gene network diagram between lncRNA genes and target genes to explore the relationship between differentially expressed lncRNAs and tumors [10].

In this study, we analyzed the differentially expressed genes of osteosarcoma cells *in situ* and in lung metastases. We found that the expression of lncRNA LOC100129620 in osteosarcoma lung metastases was significantly increased. Therefore, we examined the effect of LOC100129620 on the proliferation, apoptosis, migration, and invasion of osteosarcoma cells, detected the effect of LOC100129620 on the progression of osteosarcoma cells *in vivo*, and explored the mechanism of action of LOC100129620. Our study facilitates the identification of new tumor treatment targets.

## MATERIALS AND METHODS

### Cell culture

Human bone marrow mesenchymal stem cells (hBMSCs) were isolated from the bone marrow of patients who underwent joint replacement surgery. The collection of specimens was approved by the hospital ethics committee, and informed consent was signed by all patients. The osteosarcoma cell lines (U2OS, MG63, HOS, and Saos-2) were purchased from the Cell Bank of the Type Culture Collection Committee of the Chinese Academy of Sciences. hFOB1.19 cells were purchased from the ATCC cell bank. The cells were cultured in DMEM supplemented with 10% fetal bovine serum (FBS) (Gibco, USA) and 1% penicillin–streptomycin (Hyclone, USA).

### RNA isolation and quantitative real-time PCR

Total RNA was isolated from cells and tissue using TRIzol reagent (Thermo Fisher Scientific, USA) according to the manufacturer’s instructions. cDNA was obtained using PrimeScript™ RT Master Mix (Takara, RR036A) according to the manufacturer’s instructions. Quantitative real-time PCR (qRT-PCR) was conducted with SYBR® Premix Ex Taq (Takara, RR420A) using a Roche LightCycler 480 II fluorescence quantitative PCR instrument. The qRT-PCR programs were as follows: 95° C for 60 s, followed by 50 cycles of 95° C for 5 s and 60° C for 30 s. The data were analyzed using the 2^−ΔΔCt^ method. U6 or GAPDH was used as internal control.

### Immunofluorescence *in situ* hybridization

Immunofluorescence *in situ* hybridization was performed according to the manufacturer’s instructions. Briefly, cells were fixed with 4% paraformaldehyde (PFA) for 10 min and washed twice with absolute ethanol. The labeled fluorescent probe was put into hybridization buffer and incubated at 55° C for 30 min, and hybridization probes were added and incubated at 55° C for 16 h. Hybridization solution was replaced with fresh solution and incubated for 30 min. Glycerol was used to mount the slides for fluorescence observation.

### Cell proliferation assay

Osteosarcoma cells were seeded into 96-well cell culture plates at a density of 1000 cells per well. CCK-8 reagent (10 μl) was added into each well, and the plates were incubated for 2 h. Absorbance at 450 nm was measured with a microplate reader. Osteosarcoma cells were seeded into 6-well cell culture plates at a density of 400 cells per well and incubated for 2 weeks. Then cells were fixed with 4% PFA for 15 min and stained with 0.1% crystal violet, and pictures were taken with a scanner to calculate the number of colonies formed.

### Cell cycle detection

The cells were washed once with PBS and collected by centrifugation at 1500 rpm for 5 min. The cell concentration was adjusted to 1×10^6^ cells/ml. Of the single-cell suspension, 1 ml was centrifuged, and the supernatant was removed. Pre-cooled 70% ethanol (500 μl) was added to the cells, cells were fixed for 2 h and stored overnight at 4° C, and ethanol was washed off with PBS before staining. RNase A solution (100 μl) was added to the cell pellet, and the cells were resuspended and incubated in a water bath at 37° C for 30 min. Then 400 μl PI staining solution was added, and samples were incubated at 4° C for 30 min in the dark. Cells were analyzed by flow cytometry.

### Apoptosis assay

The cells were collected, and the cell concentration was adjusted to 1×10^6^ cells/ml. Cells were washed twice with cold PBS. The cells were resuspended in 1 ml Binding Buffer and centrifuged at 1000 rpm for 10 min, and the supernatant was discarded. The cells were resuspended in 100 μl Binding Buffer so that the cell density was 1×10^6^ cells/ml. Annexin V-FITC (5 μl) was added to the tube at room temperature avoiding light. Samples were mixed gently for 10 min. PI (5 μl) was added, and samples were incubated for 5 min at room temperature avoiding light. PBS (500 μl) was added, and samples were mixed gently. Cells were analyzed by flow cytometry within 1 h.

### Cell migration and invasion assay

For the cell migration assay, cells were inoculated in a 6-well plate. When the cells reached 100% confluency, the plates were scratched using 1 ml tips, and pictures were taken (time point 0 h). Culture medium was changed to serum-free medium, and pictures were taken after 24 h to record the migration distance of the cells. For the invasion assay, BD Matrigel (50 μl) was spread evenly on the upper layer of a Transwell chamber, which was incubated at 37° C for 45 min. Into the upper layer of each Transwell chamber, 20,000 cells were inoculated, and 2% FBS medium or cell culture supernatant was added to the lower layer of the Transwell chamber. At 24 h after cells were seeded, the cells on the upper layer of the Transwell chamber were wiped off, and the remaining cells were stained with crystal violet. Pictures were taken under a microscope to observe the number of cells.

### Xenografted tumor formation

This experiment was approved by the Ethics Committee of the Second Affiliated Hospital of Soochow University. Eight-week-old male nude mice were randomly selected. When the cells were in the logarithmic growth phase, they were collected and resuspended in serum-free medium. The cell concentration was adjusted to 1×10^7^ cells/ml, and 0.1 ml of the cell suspension was transplanted into the back of the axilla of nude mice. Lumps could be seen under the skin about a week after inoculation. The tumor size was measured with vernier calipers at 1, 2, and 3 weeks after inoculation. Tumor volume was calculated as follows: tumor volume = 0.5 × *a* × *b*^2^ (where *a* is the long axis, and *b* is the short axis). Three weeks after inoculation, the mice were killed by cervical dislocation, and the tumors were harvested and weighed.

### MS2bs RNA immunoprecipitation assay

MS2bs-MS2bp-based RNA immunoprecipitation (RIP) was performed according to a previous report (PMID: 21307942). Briefly, MS2bs-LOC100129620 or MS2bs-NC plasmid was co-transfected with MS2bp-GFP plasmid into U2OS cells. RIP was performed with anti-GFP and a Magna RIP RNA-Binding Protein Immunoprecipitation Kit after transfection for 48 h. Then the miRNA expression levels were measured by qRT-PCR.

### MiRNA pull-down assay

The miRNA pull-down assay was performed according to a previous report (PMID: 24710105). Briefly, biotinylated miRNA was transfected into U2OS cells, and the cells were harvested at 48 h post-transfection. The U2OS cell lysates were incubated with M-280 streptavidin magnetic beads. The RNA binding to magnetic beads was purified and analyzed by qRT-PCR.

### Dual-luciferase reporter gene detection

U2OS cells were seeded into 96-well plates at 70% confluence. Plasmid and miRNA were transfected into U2OS cells using lipofectamine 2000. After 4 h, medium was refreshed. At 48 h post-co-transfection, the medium was discarded, and cells were washed with 100 μl 1× PBS once. Next, 50 μl 1× PBS was added to each well, plates were shaken for 15 min at room temperature, and cells were lysed. Lysates were centrifuged, and 10 μl of the supernatant was added to each well of a white opaque 96-well microtiter plate. Pre-mixed LARII (100 μl) and pre-mixed Stop and Glo Reagent (100 μl) were added to each well, and after 2 s of inactivity, absorbance was measured with a microplate reader.

### Western blot

Cells were washed with pre-cooled PBS and lysed with RIPA lysis buffer. Lysate was centrifuged at 12,000 rpm for 10 min. The supernatant was used for further analysis. The protein concentration was determined by the BCA method. Samples were mixed with 4× SDS loading buffer and boiled for 5 min. Protein was separated by SDS-PAGE using 20 μg protein per lane and transferred to a PVDF membrane. The membrane was blocked with blocking solution (5% BSA) at room temperature for 1 h and incubated overnight at 4° C with primary antibody (diluted in blocking solution). Membranes were washed three times with TBST (10 min each), incubated with secondary antibody for 1 h at room temperature, and washed three times with TBST (about 10 min each). Protein bands were visualized using chemiluminescent substrate working solution and imaged with a BIO-RAD gel imaging system.

### Flow cytometry analysis of the percentage of M2 macrophages in xenografted osteosarcoma tumor tissue

Flow cytometry was carried out as previously described [11]. The mice were killed by cervical dislocation, and the tumor tissue was harvested, cut up, and incubated with 488 U/ml collagenase I (Sigma, #C0130), 230 U/ml collagenase Xi (Sigma, #C7657), 125 U/ml hyaluronidase (Sigma, #H3506), and 60 U/ml DNase I (Sigma, #d4527) at 37° C for 45 min. The digest was filtered by a 70 μm cell filter, and 1×10^7^ cells were stained with an FITC-conjugated F4/80 antibody from Abcam and a PE-conjugated CD206 antibody from R&D Systems and analyzed by flow cytometry.

### Statistical analysis

SPSS analysis software (SPSS 13.0) was used for statistical analysis. The results are expressed as mean ± standard deviation. The means of two samples were compared by the independent-sample Student’s *t*-test, and the means of multiple samples were compared by one-way analysis of variance (ANOVA). *P* < 0.05 was considered to indicate statistical significance.

## RESULTS

### LncRNA LOC100129620 is upregulated in osteosarcoma lung metastasis tissue and osteosarcoma cell lines

**Figure 1:**
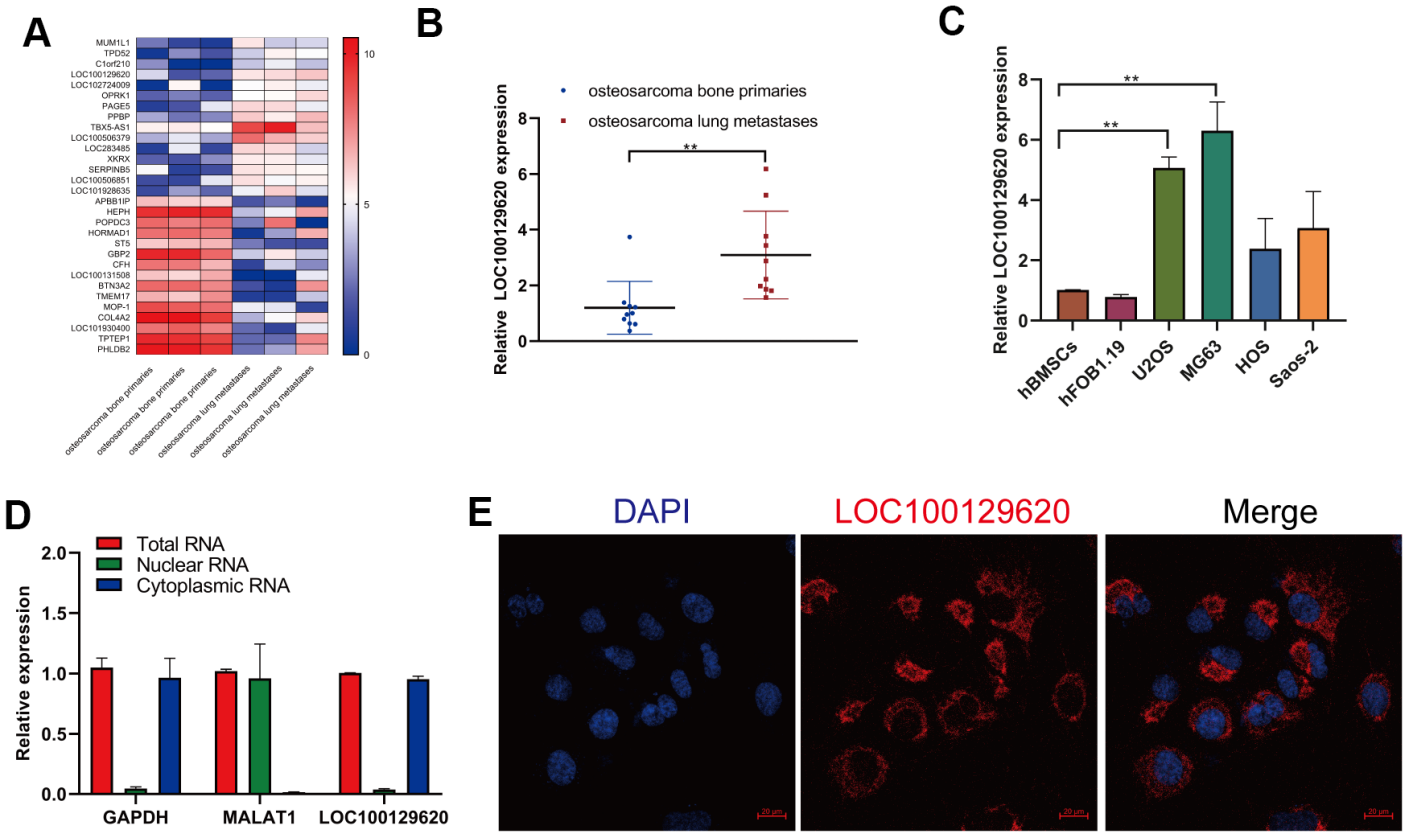
**LncRNA LOC100129620 is upregulated in osteosarcoma lung metastasis tissue and osteosarcoma cell lines.** (**A**) Heatmap analysis of the changes of gene expression in osteosarcoma bone primary tissues and osteosarcoma lung metastases. (**B**) LOC100129620 expression was analyzed by qRT-PCR in osteosarcoma bone primary tissues and osteosarcoma lung metastases. Primary tissues, *n* = 10; lung metastases, *n* = 10. (**C**) The expression of LOC100129620 in hBMSCs, hFOB1.19, and osteosarcoma cell lines (U2OS, MG63, HOS, and Saos-2) was detected by qRT-PCR. (**D**) The nuclear and cytoplasmic levels of LOC100129620 in U2OS cells were detected by qRT-PCR. GAPDH and lncRNA MALAT1 were used as controls. (**E**) The expression of LOC100129620 in U2OS cells was detected by *in situ* hybridization. Blue indicates nuclei (stained with DAPI), and red indicates LOC10012960. Scale bar, 20 μm. Statistical analysis was conducted using Student’s *t*-test. Values are means ± SD. *P < 0.05 and **P < 0.01.

In order to detect the role of gene expression in the metastasis of osteosarcoma, we first analyzed the expression data from human osteosarcoma bone primary tissues and lung metastases in GEO (dataset: GSE85537). Heatmap analysis shows that the gene expression profile in orthotopic osteosarcoma primary tissue and osteosarcoma lung metastasis tissue was significantly different (Figure 1A). The expression of TPD52, C1orf210, LOC100129620, and LOC102724009 was significantly higher in extracted lung metastasis tissue than in *in situ* osteosarcoma tissue (Figure 1B and Supplementary Figure 1A), while the expression of APBB1IP, HORMAD1, ST5, and LOC101930400 was significantly lower in lung metastasis tissue than in orthotopic osteosarcoma tissue (Supplementary Figure 1B). Then we detected the expression of LOC101930400 in hBMSCs, hFOB1.19 cells, and osteosarcoma cell lines. The expression of LOC101930400 in U2OS, MG63, HOS, and Saos-2 cells was higher than that in hBMSCs and hFOB1.19 cells (Figure 1C). The location of LOC101930400 in cells is important to its function in osteosarcoma cells. The qRT-PCR results show that LOC101930400 was mostly located in the cytoplasm, using GAPDH and lncRNA MALAT1 as controls (Figure 1D). Immunofluorescence *in situ* hybridization showed that LOC101930400 was localized in the cytoplasm; its nuclear levels were very low (Figure 1E).

### LncRNA LOC100129620 overexpression promotes the proliferation and invasion of osteosarcoma cells

**Figure 2:**
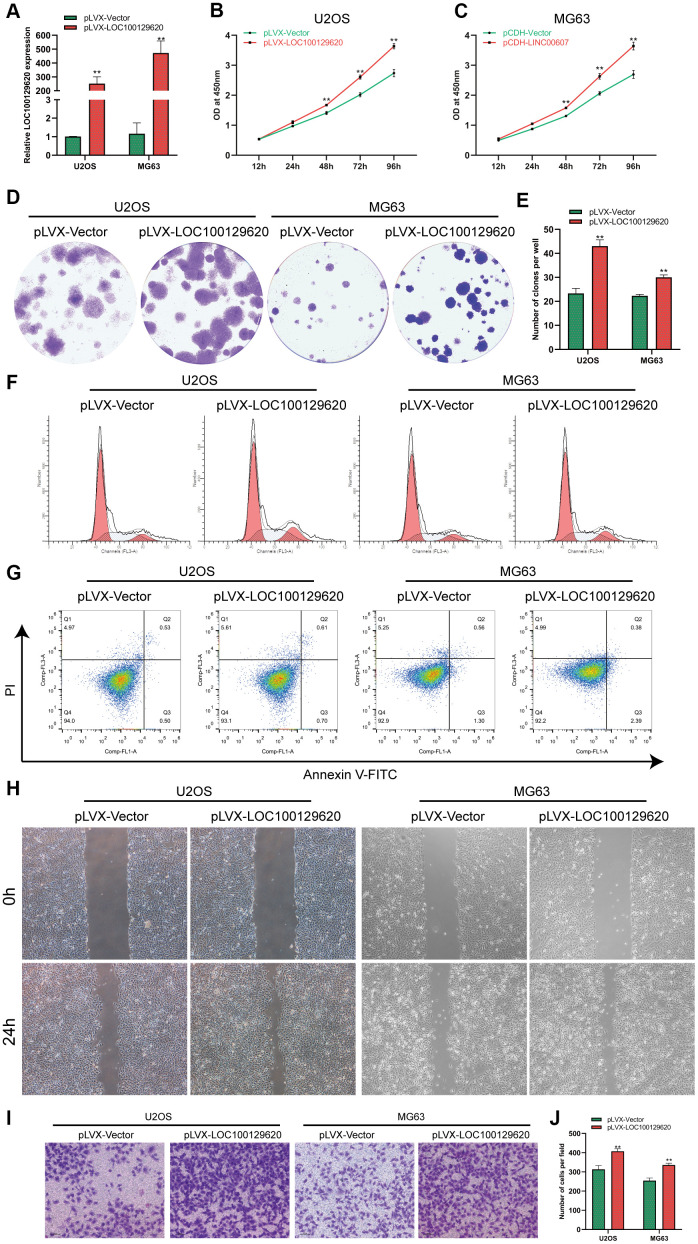
**LncRNA LOC100129620 overexpression promotes the proliferation and invasion of osteosarcoma cells.** (**A**) The expression of LOC100129620 in U2OS and MG63 cells transfected with pLVX-Vector or pLVX-LOC100129620. (**B**) CCK-8 assay to analyze the viability of U2OS cells transfected with pLVX-Vector or pLVX-LOC100129620. (**C**) CCK-8 assay to analyze the viability of MG63 cells transfected with pLVX-Vector or pLVX-LOC100129620. (**D**) Colony formation assay to analyze the proliferation of U2OS and MG63 cells transfected with pLVX-Vector or pLVX-LOC100129620. (**E**) Quantitative analysis of colony formation of U2OS and MG63 cells with transfected pLVX-Vector or pLVX-LOC100129620. (**F**) Flow cytometry analysis of the cell cycle in U2OS and MG63 cells transfected with pLVX-Vector or pLVX-LOC100129620. (**G**) Flow cytometry analysis of apoptosis of U2OS and MG63 cells transfected with pLVX-Vector or pLVX-LOC100129620. (**H**) Cell scratch assay to detect the migration ability of U2OS and MG63 cells transfected with pLVX-Vector or pLVX-LOC100129620. (**I**) Transwell assay to detect the invasion ability of U2OS and MG63 cells transfected with pLVX-Vector or pLVX-LOC100129620. (**J**) Quantitative analysis of the Transwell assay results of U2OS and MG63 cells transfected with pLVX-Vector or pLVX-LOC100129620. Statistical analysis was conducted using Student’s *t*-test. Values are means ± SD. *P < 0.05 and **P < 0.01.

The expression of LOC100129620 in U2OS and MG63 cells in the pLVX-LOC100129620 group was significantly higher than that in the pLVX-Vector group (Figure 2A). CCK-8 results show that overexpression of LOC100129620 could promote the proliferation of U2OS and MG63 cells (Figure 2B, 2C). The colony formation assay indicated that LOC100129620 overexpression could increase the proliferation of U2OS and MG63 cells (Figure 2D, 2E). Flow cytometry was used to detect the influence of LOC100129620 on the cell cycle of osteosarcoma cells. The results show that overexpression of LOC100129620 could increase the proportion of U2OS and MG63 cells in the S phase and the G2/M phase and reduce the proportion of cells in the G1 phase (Figure 2F and Supplementary Figure 2A). However, overexpression of LOC100129620 had no effect on the apoptosis of U2OS and MG63 cells (Figure 2G). The cell scratch assay showed that overexpression of LOC100129620 could promote the migration of U2OS and MG63 cells (Figure 2H). The Transwell assay results show that overexpression of LOC100129620 could promote the invasion of U2OS and MG63 cells (Figure 2I, 2J).

### Suppression of lncRNA LOC100129620 expression inhibits the proliferation and invasion of osteosarcoma cells

**Figure 3:**
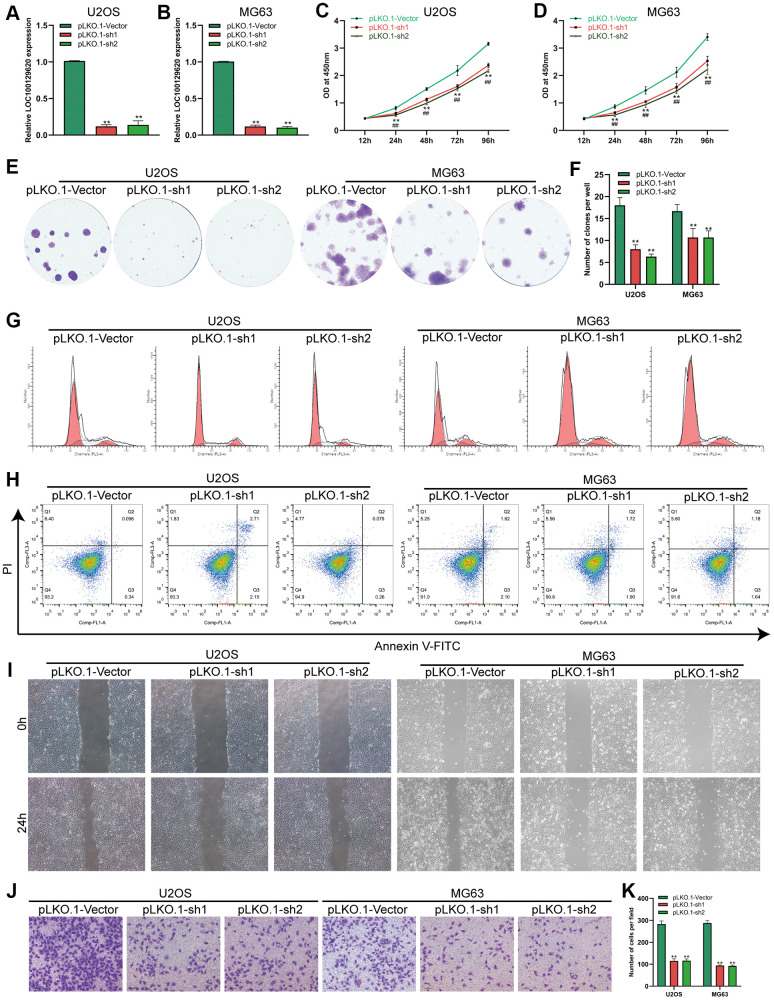
**LncRNA LOC100129620 knockdown inhibits the proliferation and invasion of osteosarcoma cells.** (**A**) The expression of LOC100129620 in U2OS cells transfected with pLKO.1-Vector, pLKO.1-sh1, or pLKO.1-sh2. (**B**) The expression of LOC100129620 in MG63 and MG63 cells transfected with pLKO.1-Vector, pLKO.1-sh1, or pLKO.1-sh2. (**C**) CCK-8 assay to analyze the viability of U2OS cells transfected with pLKO.1-Vector, pLKO.1-sh1, or pLKO.1-sh2. (**D**) CCK-8 assay to analyze the viability of MG63 cells transfected with pLKO.1-Vector, pLKO.1-sh1, or pLKO.1-sh2. (**E**) Colony formation assay to analyze the proliferation of U2OS and MG63 cells transfected with pLKO.1-Vector, pLKO.1-sh1, or pLKO.1-sh2. (**F**) Quantitative analysis of the colony formation assay results of U2OS and MG63 cells transfected with pLKO.1-Vector, pLKO.1-sh1, or pLKO.1-sh2. (**G**) Flow cytometry analysis of the cell cycle in U2OS and MG63 cells transfected with pLKO.1-Vector, pLKO.1-sh1, or pLKO.1-sh2. (**H**) Flow cytometry analysis of apoptosis of U2OS and MG63 cells transfected with pLKO.1-Vector, pLKO.1-sh1, or pLKO.1-sh2. (**I**) Cell scratch assay to detect the migration ability of U2OS and MG63 cells transfected with pLKO.1-Vector, pLKO.1-sh1, or pLKO.1-sh2. (**J**) Transwell assay to detect the invasion ability of U2OS and MG63 cells transfected with pLKO.1-Vector, pLKO.1-sh1, or pLKO.1-sh2. (**K**) Quantitative analysis of the Transwell assay results of U2OS and MG63 cells transfected with pLKO.1-Vector, pLKO.1-sh1, or pLKO.1-sh2. Statistical analysis was conducted using Student’s *t*-test. Values are means ± SD. *P < 0.05 and **P < 0.01.

The expression of LOC100129620 in U2OS and MG63 cells in the pLKO.1-sh1 and pLKO.1-sh2 groups was significantly lower than that in the pLKO.1-Vector group (Figure 3A, 3B). CCK-8 assay results show that suppression of LOC100129620 expression inhibited the proliferation of U2OS and MG63 cells (Figure 3C, 3D). The colony formation assay results indicate that suppression of LOC100129620 expression inhibited the proliferation of U2OS and MG63 cells (Figure 2E, 2F). Flow cytometry was used to detect the influence of LOC100129620 on the cell cycle of osteosarcoma cells. The results show that suppression of LOC100129620 expression could decrease the proportion of U2OS and MG63 cells in the S phase and the G2/M phase and increase the proportion of cells in the G1 phase (Figure 2G and Supplementary Figure 2B). However, suppression of LOC100129620 expression had no effect on the apoptosis of U2OS and MG63 cells (Figure 2H). The cell scratch assay showed that suppression of LOC100129620 expression inhibited the migration of U2OS and MG63 cells (Figure 2I). The Transwell assay results show that suppression of LOC100129620 expression inhibited the invasion of U2OS and MG63 cells (Figure 2J, 2K).

### LncRNA LOC100129620 regulates osteosarcoma progression *in vivo*

**Figure 4:**
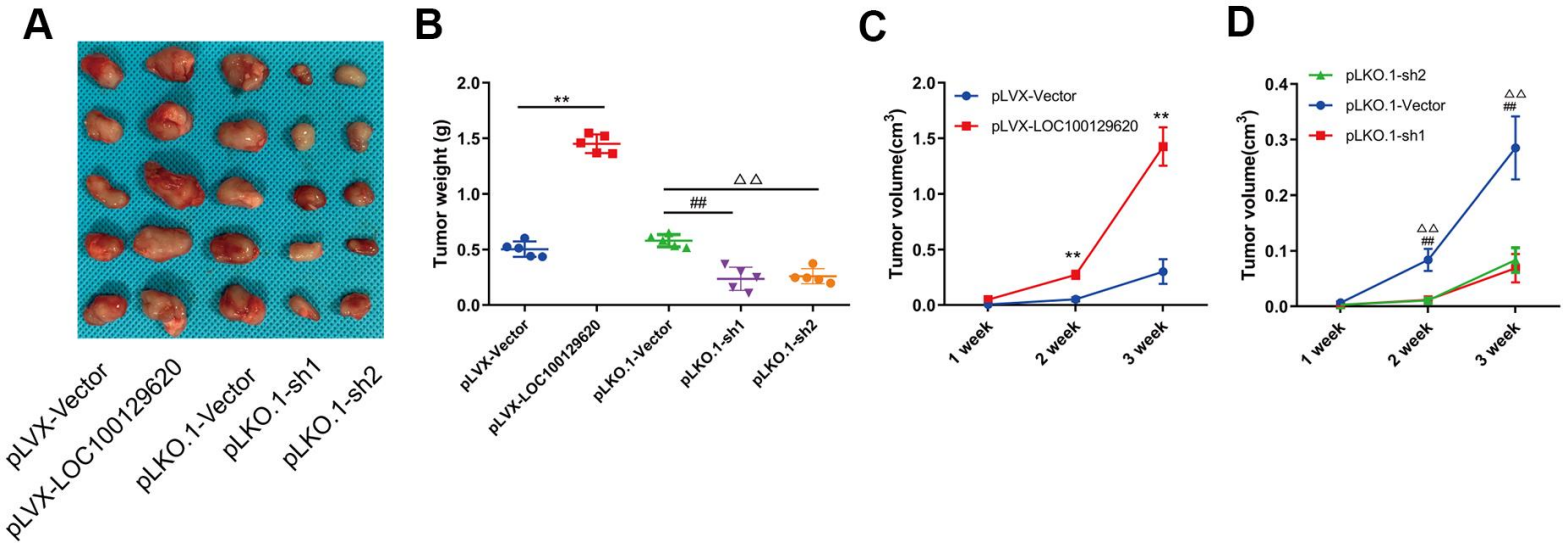
**LncRNA LOC100129620 regulates osteosarcoma progression *in vivo*.** (**A**) U2OS cells with LOC100129620 overexpression or knockdown were transplanted subcutaneously into nude mice (*n* = 5). (**B**) Tumor weight of osteosarcoma with LOC100129620 overexpression or knockdown. (**C**) Tumor weight of osteosarcoma with LOC100129620 overexpression. (**D**) Tumor weight of osteosarcoma with LOC100129620 knockdown. Statistical analysis was conducted using Student’s *t*-test. Values are means ± SD. **p < 0.01, pLVX-Vector group compared with pLVX-LOC100129620 group; ^##^*P* < 0.01, pLKO.1-Vector group compared with pLKO.1-sh1 group; ^ΔΔ^*P* < 0.01, pLKO.1-Vector group compared with pLKO.1-sh1 group.

In order to detect the effect of LOC100129620 on osteosarcoma cells *in vivo*, LOC100129620 was overexpressed or knocked down in U2OS cells, which were subsequently implanted subcutaneously in nude mice. The tumor volume was measured at 1, 2, and 3 weeks after implantation. After 3 weeks, the mice were sacrificed, and the tumors were weighed. Overexpression of LOC100129620 in U2OS cells promoted tumor growth, while LOC100129620 knockdown in U2OS cells inhibited tumor growth (Figure 4A). Tumor weight in the pLVX-LncRNA LOC100129620 group was higher than that in the pLVX-Vector group, and tumor weight in the pLKO.1-sh1 and pLKO.1-sh2 groups was lower than that in the pLKO.1-Vector group (Figure 4B). The progression of osteosarcoma cells in the pLVX-LOC100129620 group was increased compared with the pLVX-Vector group (Figure 4C). However, the progression of osteosarcoma cells in the pLKO.1-sh1 and pLKO.1-sh2 groups was decreased compared with the pLKO.1-Vector group (Figure 4D).

### LncRNA LOC100129620 regulates the function of miR-335-3p in osteosarcoma cells

**Figure 5:**
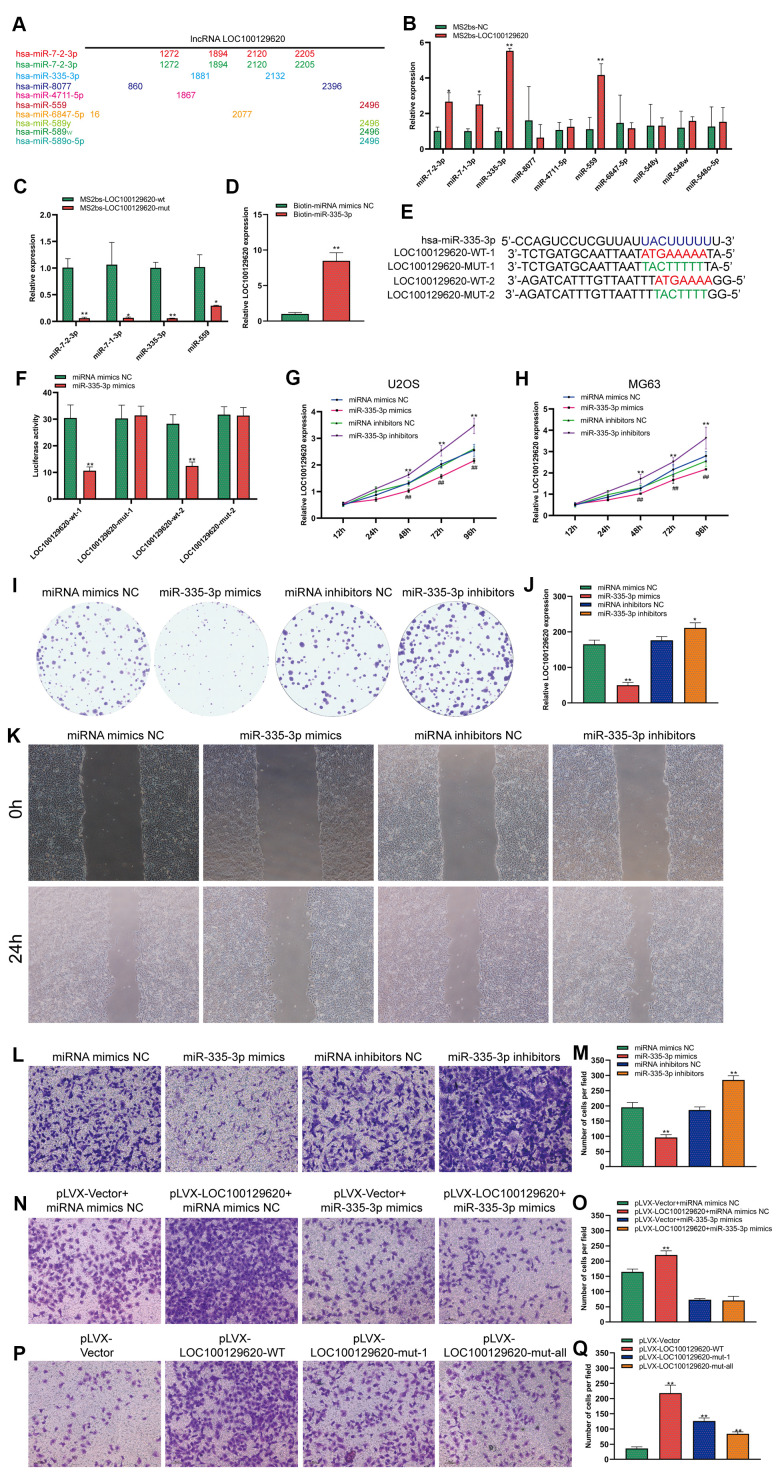
**LncRNA LOC100129620 regulates the function of miR-335-3p in osteosarcoma cells.** (**A**) MiRDB software predicted the miRNAs that could bind to LOC100129620 and the position of the binding sites. (**B**) MS2bs RNA pull-down assay to detect the miRNAs that bind to LOC100129620. (**C**) MS2bs RNA pull-down assay to detect the combination of miRNA with wild-type or mutant LOC100129620. (**D**) MiR-335-3p pull-down assay to detect the binding of miR-335-3p with LOC100129620. (**E**) Schematic diagram of the wild-type and mutated binding sites of LOC100129620 with miR-335-3p. (**F**) The binding of LOC100129620 and miR-335-3p as detected by luciferase reporter gene assay. (**G**) CCK-8 assay to detect the viability of U2OS cells treated with miR-335-3p mimics or miR-335-3p inhibitors. (**H**) CCK-8 assay to detect the viability of MG63 cells treated with miR-335-3p mimics or miR-335-3p inhibitors. (**I**) Colony formation assay to detect the viability of U2OS cells treated with miR-335-3p mimics or miR-335-3p inhibitors. (**J**) Quantitative analysis of colony formation assay results of U2OS cells treated with miR-335-3p mimics or miR-335-3p inhibitors. (**K**) Cell scratch assay to detect the migration ability of U2OS cells treated with miR-335-3p mimics or miR-335-3p inhibitors. (**L**) Transwell assay to detect the invasion ability of U2OS cells treated with miR-335-3p mimics or miR-335-3p inhibitors. (**M**) Quantitative analysis of the Transwell assay results of U2OS cells treated with miR-335-3p mimics or miR-335-3p inhibitors. (**N**) Transwell assay to detect the effect of LOC100129620 on the invasion ability of U2OS cells treated with miR-335-3p mimics or miR-335-3p inhibitors. (**O**) Quantitative analysis to detect the effect of LOC100129620 on the invasion ability of U2OS cells treated with miR-335-3p mimics or miR-335-3p inhibitors. (**P**) Transwell assay to detect the effects of wild-type LOC100129620 and miR-335-3p binding site mutant LOC100129620 on the invasion ability of U2OS cells. (**Q**) Quantitative analysis of the Transwell assay results to detect the effects of wild-type LOC100129620 and miR-335-3p binding site mutant LOC100129620 on the invasion ability of U2OS cells. Statistical analysis was conducted using Student’s *t*-test. Values are means ± SD. *P < 0.05 and **P < 0.01.

LOC100129620 is mainly located in the cytoplasm. LncRNA also affects the expression of its target genes by controlling the expression of miRNA. In some tumor cells and specific tissues, some lncRNAs carry the “seed sequence” of certain miRNAs, which bind miRNAs like sponges, thereby preventing miRNAs from binding to their target mRNAs. We used miRDB software to list the top 10 miRNAs that have binding sites to LOC100129620, and marked the location of the binding sites (Figure 5A). Subsequently, the MS2bs RIP method was used to detect the binding of LOC100129620 to miRNA. LOC100129620 can bind to miR-7-2-3p, miR-7-1-3p, miR-335-3p, and miR-559 (Figure 5B). Among these, miR-335-3p has the strongest binding affinity with LOC100129620. After we mutated the binding site of LOC100129620 with miRNA, the binding affinity was significantly reduced (Figure 5C). We also used a Biotin-miRNA pull-down assay to detect the binding of miR-335-3p and LOC100129620. The results show that miR-335-3p can bind to LOC100129620 (Figure 5D). There are two predicted binding sites for miR-335-3p in LOC100129620. Based on the predicted binding sites, we constructed binding site mutant and wild-type dual-luciferase reporter genes (Figure 5E). The detection of luciferase activity revealed that miR-335-3p can inhibit the expression of the wild-type luciferase reporter gene without affecting the activity of mutant luciferase (Figure 5F). No studies have reported the effect of miR-335-3p on the function of osteosarcoma cells. The CCK-8 assay revealed that overexpression of miR-335-3p could reduce the viability of U2OS and MG63 cells, while inhibition of miR-335-3p expression can promote the viability of U2OS and MG63 cells (Figure 5G, 5H). The results of the colony formation assay show that overexpression of miR-335-3p could effectively inhibit the proliferation of U2OS cells, while inhibition of miR-335-3p can effectively promote the proliferation of U2OS cells (Figure 5I, 5J). The results of the wound healing assay show that overexpression of miR-335-3p could effectively inhibit the migration of U2OS cells, while knockdown of miR-335-3p could effectively promote the migration of U2OS cells (Figure 5K). The results of the Transwell assay show that overexpression of miR-335-3p could effectively inhibit the invasion of U2OS cells, while inhibition of miR-335-3p could effectively promote the invasion of U2OS cells (Figure 5L, 5M). LOC100129620 could promote the invasion of U2OS cells; however, the stimulatory effects on the invasion of U2OS cells were reduced when U2OS cells overexpressed miR-335-3p (Figure 5N, 5O). However, when we mutated the binding site of LOC100129620 for miR-335-3p, the stimulatory effect of LOC100129620 on U2OS cell invasion was lost (Figure 5P, 5Q).

### LncRNA LOC100129620 regulates the expression of CDK6 through miR-335-3p

**Figure 6:**
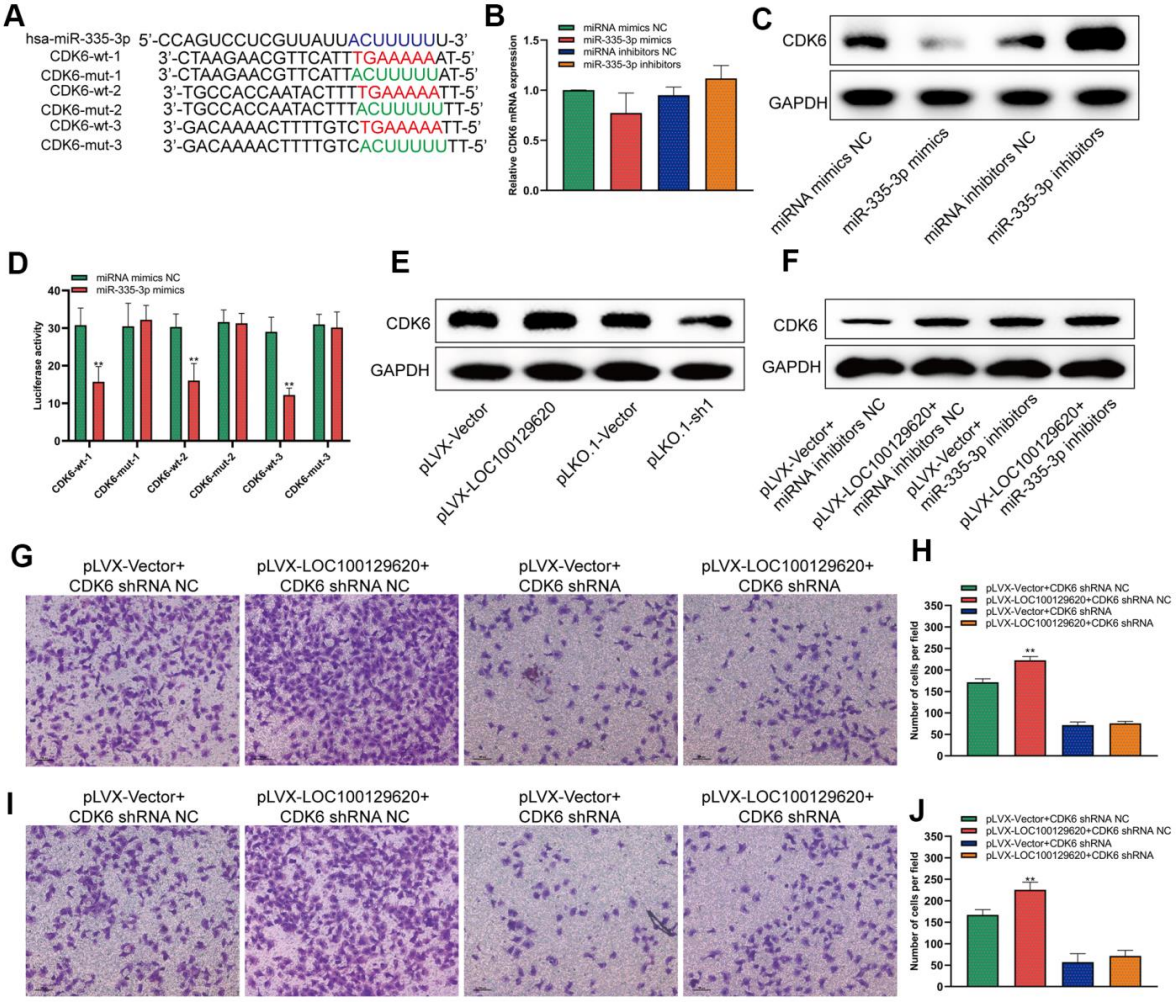
**LncRNA LOC100129620 regulates the expression of CDK6 through miR-335-3p.** (**A**) Schematic diagram of the wild-type and mutated binding sites of the CDK6 3′-UTR and miR-335-3p. (**B**) CDK6 mRNA expression in U2OS cells treated with miR-335-3p mimics or inhibitors, as detected by qRT-PCR. (**C**) Western blot to detect the effect of miR-335-3p on the protein expression of CDK6. (**D**) Binding of the CDK6 3′-UTR and miR-335-3p, as detected by luciferase reporter gene assay. (**E**) Western blot to detect the effect of LOC100129620 on the protein expression of CDK6. (**F**) Western blot to detect the effect of LOC100129620 on the protein expression of CDK6 in U2OS cells treated with miR-335-3p inhibitors. (**G**) Transwell assay to detect the effect of LOC100129620 on the invasion ability of U2OS cells with CDK6 knockdown. (**H**) Quantitative analysis of the Transwell assay results to detect the effect of LOC100129620 on the invasion ability of U2OS cells with CDK6 knockdown. (**I**) Transwell to detect the effect of LOC100129620 on the invasion ability of MG63 cells with CDK6 knockdown. (**J**) Quantitative analysis of the Transwell assay results to detect the effect of LOC100129620 on the invasion ability of MG63 cells with CDK6 knockdown. Statistical analysis was conducted using Student’s *t*-test. Values are means ± SD. **P < 0.01.

MiRNA regulates the translation of mRNA by binding to the 3′-UTR region of mRNA. It was predicted by miRDB software that miR-335-3p could bind to the 3′-UTR region of CDK6 with three different binding sites. We constructed different binding site mutation sequences (Figure 6A). Using miR-335-3p mimics and miR-335-3p inhibitors to regulate the expression of miR-335-3p in U2OS cells, overexpression or knockdown of miR-335-3p had no effect on the mRNA levels of CDK6 in U2OS cells (Figure 6B). Overexpression of miR-335-3p could inhibit the protein expression of CDK6, while knockdown of miR-335-3p could promote the protein expression of CDK6 (Figure 6C). The results of the luciferase reporter gene assay show that overexpression of miR-335-3p can inhibit the activity of the wild-type luciferase reporter gene, but has no significant effect on the activity of the mutant luciferase reporter gene (Figure 6D). Overexpression of LOC100129620 could promote the protein expression of CDK6, while inhibiting the expression of LOC100129620 could reduce the protein expression of CDK6 (Figure 6E). In osteosarcoma tissue, the expression of LOC100129620 was negatively correlated with the expression of miR-335-3p (Supplementary Figure 3A), while the expression of LOC100129620 was positively correlated with the expression of CDK6 (Supplementary Figure 3B). However, when miR-335-3p inhibitors were used to inhibit the function of miR-335-3p in U2OS cells, the stimulatory effect of LOC100129620 on CDK6 protein expression was decreased (Figure 6F). Upon CDK6 knockdown in U2OS cells, the stimulatory effects of LOC100129620 on the invasion of U2OS cells were reduced (Figure 6G, 6H). The same result was obtained in MG63 cells (Figure 6I, 6J).

### LncRNA LOC100129620 regulates osteosarcoma-induced angiogenesis

**Figure 7:**
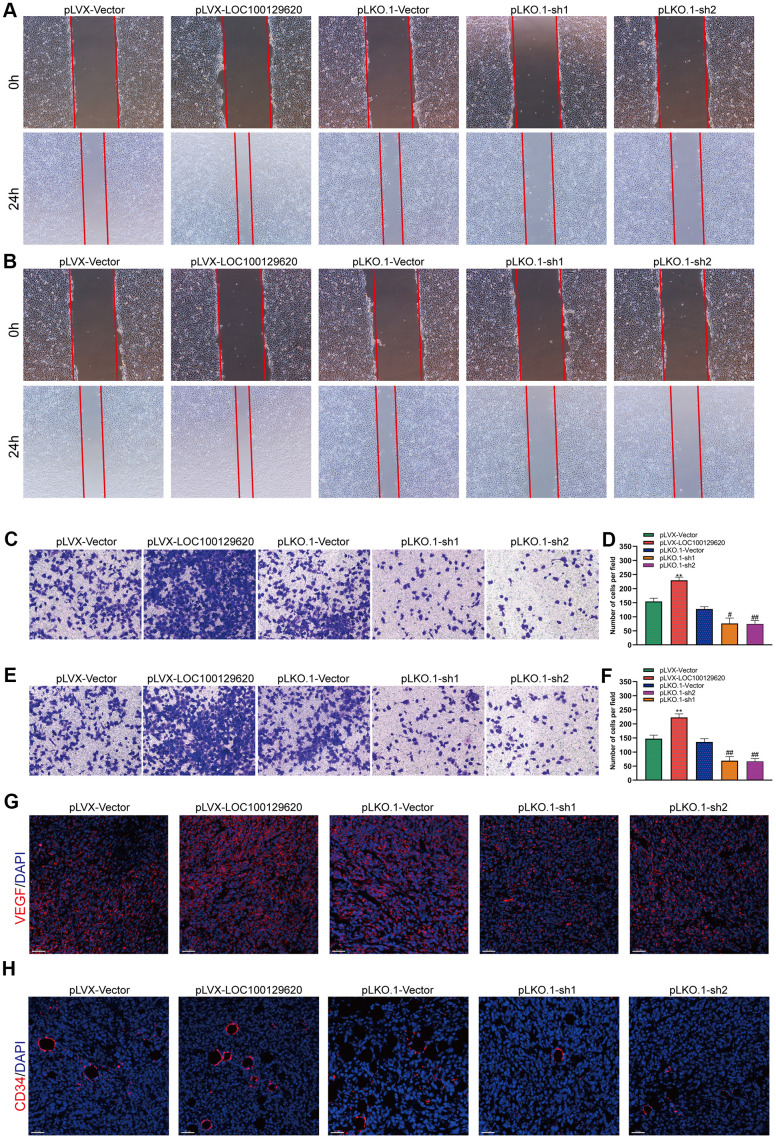
**LncRNA LOC100129620 regulates osteosarcoma-induced angiogenesis.** (**A**) Cell scratch assay to detect the migration ability of HUVECs treated with cell culture supernatant from U2OS cells with LOC100129620 overexpression or knockdown. (**B**) Cell scratch assay to detect the migration ability of HUVECs treated with cell culture supernatant isolated from MG63 cells with LOC100129620 overexpression or knockdown. (**C**) Transwell assay to detect the invasion ability of HUVECs treated with cell culture supernatant isolated from U2OS cells with LOC100129620 overexpression or knockdown. (**D**) Quantitative analysis of the Transwell assay results of HUVECs treated with cell culture supernatant isolated from U2OS cells with LOC100129620 overexpression or knockdown. (**E**) Transwell assay to detect the invasion ability of HUVECs treated with cell culture supernatant isolated from MG63 cells with LOC100129620 overexpression or knockdown. (**F**) Quantitative analysis of the Transwell assay results of HUVECs treated with cell culture supernatant isolated from MG63 cells with LOC100129620 overexpression or knockdown. Statistical analysis was conducted using Student’s *t*-test. (**G**) Immunofluorescence analysis to detect the expression of VEGF in xenograft tumor. (**H**) Immunofluorescence analysis to detect the expression of CD34 in xenograft tumor. Values are means ± SD. **P < 0.01, pLVX-Vector group compared with pLVX-LOC100129620 group; ^##^*P* < 0.01, pLKO.1-Vector group compared with pLKO.1-sh1 and pLKO.1-sh2 groups.

The formation of blood vessels inside tumors plays an important role in tumor growth. Tumor blood vessels provide tumor tissue with oxygen and nutrients necessary for its metabolism, so that tumors could grow rapidly, and at the same time provide a transport pathway for tumor metastasis. Tumor cells could promote angiogenesis by secreting a variety of cytokines. Using the culture supernatant of osteosarcoma cells to culture HUVEC cells, the effect of tumor cells on HUVECs could be observed. The results of the cell scratch experiments show that overexpression of LOC100129620 increased the stimulatory effect of osteosarcoma cells on the migration of endothelial cells, while LOC100129620 knockdown could reduce the stimulatory effects of osteosarcoma cells on the migration of endothelial cells (Figure 7A). The Transwell assay results show that overexpression of LOC100129620 increased the stimulatory effects of U2OS cells on the invasion of endothelial cells, while LOC100129620 knockdown reduced the stimulatory effects of U2OS cells on the invasion of endothelial cells (Figure 7B, 7C). The same results were obtained in MG63 cells (Figure 7E, 7F). Immunofluorescence analysis showed that overexpression of LOC100129620 promoted the expression of VEGF, while knockdown of LOC100129620 inhibited the expression of VEGF within the xenograft model (Figure 7G). Similarly, overexpression of LOC100129620 increased the number of endothelial cells, while knockdown of LOC100129620 reduced the number of endothelial cells within the xenograft model (Figure 7H).

### LncRNA LOC100129620 regulates osteosarcoma-associated macrophage polarization

**Figure 8:**
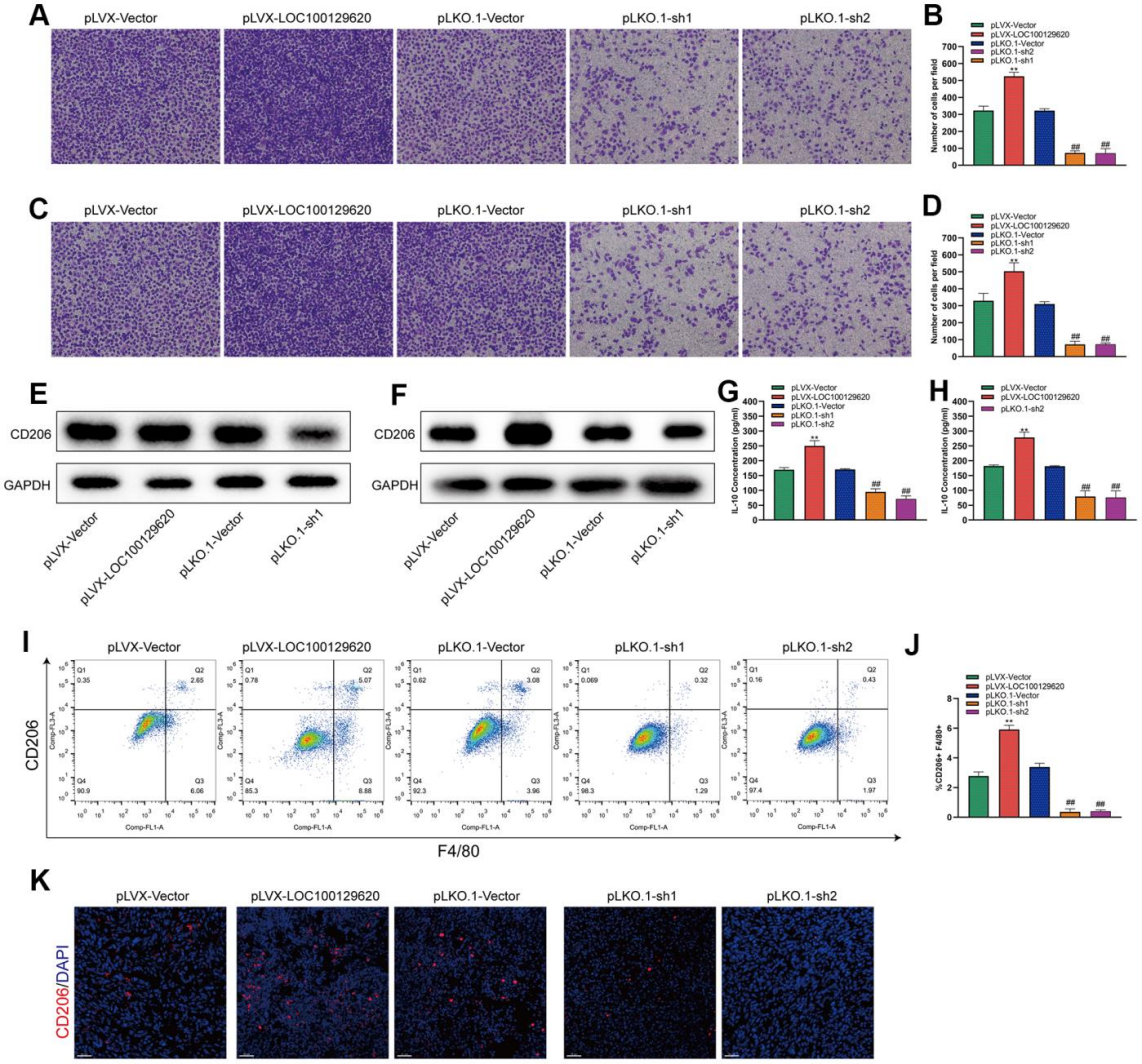
**LncRNA LOC100129620 regulates osteosarcoma-associated macrophage polarization.** (**A**) Transwell assay to detect the invasion ability of RAW264.7 cells treated with cell culture supernatant isolated from U2OS cells with LOC100129620 overexpression or knockdown. (**B**) Quantitative analysis of the Transwell assay results of RAW264.7 cells treated with cell culture supernatant isolated from U2OS cells with LOC100129620 overexpression or knockdown. (**C**) Transwell assay to detect the invasion ability of RAW264.7 cells treated with cell culture supernatant isolated from MG63 cells with LOC100129620 overexpression or knockdown. (**D**) Quantitative analysis of the Transwell assay results of RAW264.7 cells treated with cell culture supernatant isolated from MG63 cells with LOC100129620 overexpression or knockdown. (**E**) Western blot to detect the expression of CD206 in RAW264.7 cells treated with cell culture supernatant isolated from U2OS cells with LOC100129620 overexpression or knockdown. (**F**) Western blot to detect the expression of CD206 in RAW264.7 cells treated with cell culture supernatant isolated from MG63 cells with LOC100129620 overexpression or knockdown. (**G**) ELISA to detect the expression of IL-10 in cell culture supernatant isolated from U2OS cells with LOC100129620 overexpression or knockdown. (**H**) ELISA to detect the expression of IL-10 in cell culture supernatant isolated from MG63 cells with LOC100129620 overexpression or knockdown. (**I**) The percentages of total (F4/80+) and M2 (CD206+) macrophages in the tumor tissues were assayed by flow cytometry. (**J**) Statistical analysis of the percentages of total (F4/80+) and M2 (CD206+) macrophages in the tumor tissues. (**K**) Immunofluorescence analysis of the percentage of M2 (CD206+) macrophages in the tumor tissues. Statistical analysis was conducted using Student’s *t*-test. Values are means ± SD. **P < 0.01, pLVX-Vector group compared with pLVX-LOC100129620 group; ^##^*P* < 0.01, pLKO.1-Vector group compared with pLKO.1-sh1 and pLKO.1-sh2 groups.

Tumor-associated macrophages (TAMs) play an important role in the tumor microenvironment. Two subtypes that can polarize each other exist: the M1 type (classically activated macrophages) and the M2 type (alternatively activated macrophages). M1 type TAMs play a role in inhibiting tumor growth, while M2 type TAMs promote and support the occurrence and development of tumors. In a specific tumor microenvironment, M2 type TAMs occupy a dominant position and promote tumor development. The Transwell assay results show that overexpression of LOC100129620 could increase the stimulatory effect of U2OS cells on the invasion of RAW264.7 cells, while knockdown of LOC100129620 could reduce the stimulatory effect of RAW264.7 cells on the invasion of endothelial cells (Figure 8A, 8B). Similar results were obtained in MG63 cells (Figure 8C, 8D). Overexpression of LOC100129620 in U2OS cells promoted the expression of CD206, which is an important marker for M2 type TAMs in RAW264.7 cells; however, LOC100129620 knockdown in U2OS inhibited the expression of CD206 in RAW264.7 cells (Figure 8E). Similar results were obtained in MG63 cells (Figure 8F). LOC100129620 overexpression promoted IL-10 expression in osteosarcoma cells, and LOC100129620 knockdown inhibited IL-10 expression in osteosarcoma cells (Figure 8G, 8H). To extend our *in vitro* observations, we investigated whether LOC100129620 could regulate the infiltration and M2 polarization of macrophages, and thus facilitate osteosarcoma cell growth, *in vivo*. Flow cytometry analysis showed that LOC100129620 overexpression promoted the infiltration and M2 polarization of macrophages in osteosarcoma tumor tissue, and LOC100129620 knockdown reduced the infiltration and M2 polarization of macrophages in osteosarcoma tumor tissue (Figure 8I, 8J).

## DISCUSSION

Osteosarcoma is a malignant bone tumor that mainly affects children and adolescents, and its treatment has always been a clinical challenge. The main treatment strategy for osteosarcoma is surgery combined with neoadjuvant chemotherapy. Once osteosarcoma metastasizes, especially to the lungs, the long-term survival rate is less than 30%. Therefore, inhibiting the metastasis of osteosarcoma is the focus of reducing the mortality of patients with osteosarcoma. In order to characterize the biological mechanism leading to lung metastasis of osteosarcoma cells, we analyzed the difference in gene expression in orthotopic osteosarcoma tissue and osteosarcoma lung metastasis tissue. The results show that there is a large difference in gene expression between *in situ* osteosarcoma tissues and lung metastases from osteosarcoma, including many lncRNAs. Analysis of an increased number of samples revealed that the expression of LOC100129620 in osteosarcoma lung metastasis tissues was significantly higher than that in osteosarcoma *in situ* tissue. Some studies have reported the effect of lncRNA on osteosarcoma cells. LncRNA HIF1A-AS2 promotes the progression of osteosarcoma by regulating the function of miR-129-5p [12]. LncRNA KCNQ1OT1 functions as a competing endogenous RNA (ceRNA) for miR-4458, regulating the expression of CCND2 and enhancing the progression of osteosarcoma [13]. LncRNA DLEU1 inhibits osteosarcoma progression by regulating the function of the miR-671-5p/DDX5 axis [14]. However, the regulatory effect of LOC100129620 on osteosarcoma cells has not been reported yet. The study of lncRNAs has shown that they can perform biological functions in the nucleus or in the cytoplasm. Under normal circumstances, lncRNA mainly regulates chromatin architecture, transcription regulation, and alternative splicing when located in the nucleus; when located in the cytoplasm, lncRNA mainly functions through ceRNA regulatory mechanisms, adsorbing miRNA and affecting mRNA stability and translation [15–17]. The subcellular location of lncRNA in tumor cells determines its function. Through qRT-PCR and immunofluorescence *in situ* hybridization, we found that LOC100129620 is mainly located in the cytoplasm. By overexpressing and knocking down LOC100129620 in osteosarcoma cells, we found that it can promote the proliferation, migration, and invasion of osteosarcoma cells and promote the progression of osteosarcoma *in vivo*.

Using bioinformatics software analysis and experimental verification, we found that LOC100129620 regulates the function of miR-335-3p through sponge adsorption and thus regulates the expression of CDK6 through miR-335-3p. At present, no studies have reported the regulatory effect of miR-335-3p on osteosarcoma cells. Our results indicate that overexpression of miR-335-3p could effectively inhibit the proliferation, migration, and invasion of osteosarcoma cells, while miR-335-3p knockdown could promote the proliferation, migration, and invasion of osteosarcoma cells. Therefore, miR-335-3p is an inhibitor of osteosarcoma progression. The study of the relationship between cell cycle regulation and tumorigenesis is also an important focus of tumor research in recent years [18]. CDK6, a member of the cyclin-dependent kinase (CDK) family, is an important regulatory factor in the G1 phase of the cell cycle [19]. CDK6 has an important regulatory role in the intracellular signal network, and it can affect important processes such as the cell cycle, differentiation, and apoptosis [20].

Tumor angiogenesis plays an important role in tumor occurrence and development, and has become a hot topic in the field of tumors in recent years [21, 22]. Angiogenesis refers to the development of new blood vessels from existing capillaries or post-capillary veins. In general, angiogenic factors and vascular inhibitors coordinate the entire process of angiogenesis in tissues. Under the influence of external factors, internal gene mutations, tumorigenesis, and other conditions, angiogenic factors are over-activated, while vascular inhibitory factors are inhibited. The imbalance of the two can activate the angiogenesis system and cause tissues to overgenerate blood vessels. The rapid vascularization facilitates tumor growth and proliferation and metastasis of tumor cells. In this study, we found that LOC100129620 could regulate tumor cell-mediated angiogenesis.

TAMs are an important part of the tumor microenvironment [23]. They play a key role in tumor-related inflammation and promote tumor development and metastasis [24]. They are now considered to be an indispensable factor for tumor progression and immune escape [25]. TAMs are characterized by their heterogeneity and plasticity. Under the stimulation of different factors, macrophages can produce different polarization types. Tumor cytokines, chemokines, microbial infections, and even drug intervention can reprogram macrophages to a functionally polarized phenotype. According to their activation state, activated receptor phenotype, and function, macrophages can be divided into two subtypes: M1 and M2. In this study, we found that LOC100129620 can promote the polarization of TAMs to the M2 type.

## CONCLUSIONS

In this study, we found that LOC100129620 promotes the proliferation, migration, and invasion of osteosarcoma cells through the LOC100129620/miR-335-3p/CDK6 signaling axis. At the same time, LOC100129620 can promote the regeneration of blood vessels and the polarization of macrophages.

## Supplementary Material

Supplementary Figures
