# Supplementary material for: LncRNA LOC100129620 promotes osteosarcoma progression through regulating CDK6 expression, tumor angiogenesis, and macrophage polarization
Source: Aging (Albany NY). 2021 May 18;13(10):14258–76. doi: 10.18632/aging.203042 (PMC8202873; doi:10.18632/aging.203042)
Supplement: Supplementary Figures [file aging-13-203042-s001.pdf]

SUPPLEMENTARY FIGURES

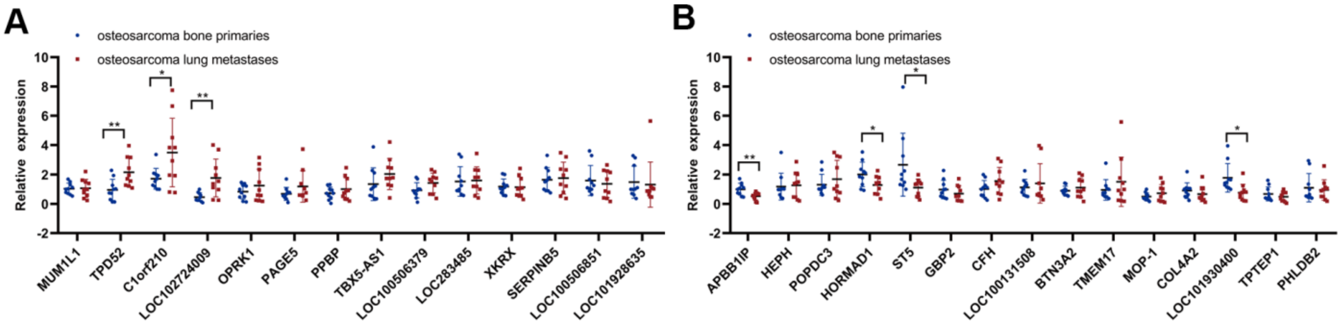

Supplementary Figure 1. LncRNA and mRNA expression in osteosarcoma bone primary tissues and osteosarcoma lung metastases. (**A**) Upregulated lncRNAs and mRNAs in osteosarcoma bone primary tissues and osteosarcoma lung metastases were detected by qRT-PCR and are depicted in a heatmap. Osteosarcoma bone primary tissues, *n* = 10; osteosarcoma lung metastases, *n* = 10. (**B**) Downregulated lncRNAs and mRNAs in osteosarcoma bone primary tissues and osteosarcoma lung metastases were detected by qRT-PCR and are depicted in a heatmap. Osteosarcoma bone primary tissues, *n* = 10; osteosarcoma lung metastases, *n* = 10. Statistical analysis was conducted using Student's *t*-test. Values are means ± SD. **P* < 0.05 and ***P* < 0.01.

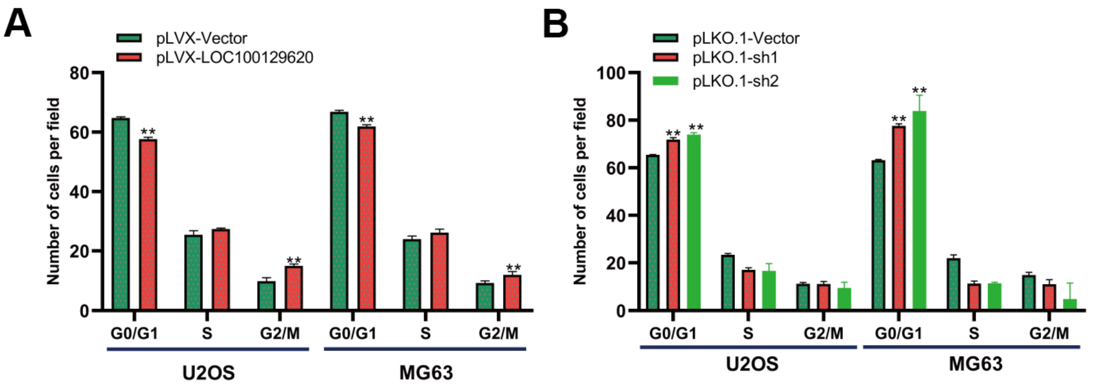

Supplementary Figure 2. LncRNA LOC100129620 regulates the cell cycle of osteosarcoma cells. (**A**) Quantitative flow cytometry analysis of the cell cycle of U2OS and MG63 cells transfected with pLVX-Vector and pLVX-LOC100129620. (**B**) Quantitative flow cytometry analysis of the cell cycle of U2OS and MG63 cells transfected with pLKO.1-Vector, pLKO.1-sh1, or pLKO.1-sh2. Statistical analysis was conducted using Student's *t*-test. Values are means ± SD. ***P* < 0.01.

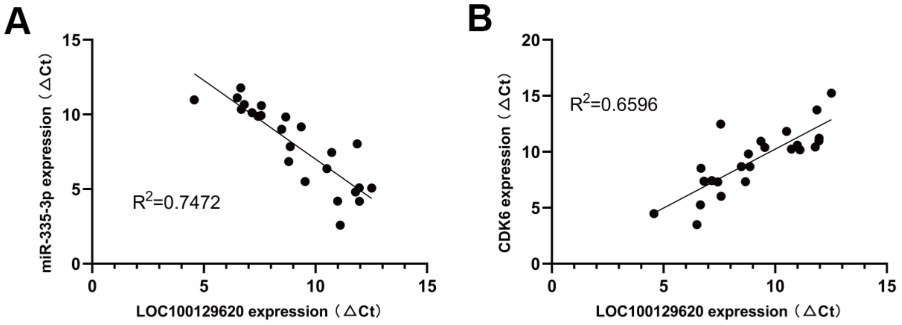

Supplementary Figure 3. The correlation between LOC100129620 and miR-335-3p (**A**) or CDK6 (**B**) in osteosarcoma tissue.
